# Antibacterial and Antifungal Activities of *Musa balbisiana* Parts

**DOI:** 10.1002/fsn3.70732

**Published:** 2025-07-28

**Authors:** Hoang Thi Ngoc Nhon, Dinh Khanh Dieu, Dong Thi Anh Dao, Le Thi Hong Anh

**Affiliations:** ^1^ Ho Chi Minh City University of Industry and Trade (HUIT) Ho Chi Minh City Vietnam; ^2^ Ho Chi Minh City University of Technology (HCMUT) Ho Chi Minh City Vietnam; ^3^ Vietnam National University Ho Chi Minh City Ho Chi Minh City Vietnam

**Keywords:** antibacterial, antifungal, bioactive compounds, *Musa balbisiana*

## Abstract

*Musa balbisiana* parts contain various secondary metabolites with significant biological activities, including antibacterial and antifungal properties. This study aimed to evaluate the antibacterial properties of different parts of *M. balbisiana* against four selected strains with two positive‐gram ( *Staphylococcus aureus* , *Bacillus subtilis* ) and two negative‐gram ( *Escherichia coli* , *Salmonella enterica* ) as well as the antifungal ability of two strains of *Aspergillus niger* and *Aspergillus flavus* of *M. balbisiana* parts. The results showed that *M. balbisiana* parts reveal potential antibacterial and antifungal activities. The investigated samples inhibit positive‐gram bacteria better than negative‐gram bacteria. The seed sample demonstrated the strongest antibacterial effect compared to other parts in both MBC and MIC assays. Moreover, the seed sample also indicated the highest anti‐mold ability on *A. niger* and *A. flavus* among the six investigated parts. The findings offer the platform for further applied studies using these parts of *M. balbisiana,* especially the seed sample in antibacterial and anti‐mold products.

## Graphical abstract

The antibacterial and antimould activities of *Musa balbisiana* parts were concentration‐dependent. Seed sample indicated the highest ability to have both studied bioactive properties among the six selected parts. *M. balbisiana* parts pose potential antibacterial via two antibacterial activity assays of (MIC and MBC) on four investigated bacteria (*Staphylococcus aureus*, *Bacillus subtilis*, *Escherichia coli*, *Salmonella enterica*). The samples could inhibit both *Aspergillus niger* and *Aspergillus flavus*, with the inhibition ability of *A. niger* being better than *A. flavus*.

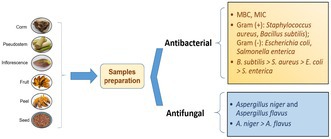

## 1 Introduction

*Musa balbisiana* is a wild species of the *Musa* genus, widely distributed across Asia, including Vietnam (Scot et al. 2006). Owing to its rich content of bioactive compounds, various parts of this plant have indicated various biological activities and effects, including antioxidant, antibacterial, antivirus, anti‐allergic, anti‐inflammatory, etc. (Cook 1996). Traditionally, different parts of *M. balbisiana* have been used in folk medicine as the remedy for many disorders related to toothache, diabetes, wounds, eyes, digestive system, etc. (Bich 2014; Pereira and Maraschin 2015). These therapeutic effects are attributed to the presence of bioactive constituents such as saponins, polyphenols, alkaloids, flavonoids, etc. Thus, this plant represents a promising natural source for the development of plant‐based therapeutic agents. Both phytochemicals and plant extracts have known antimicrobial and antifungal properties, making them highly relevant for modern medicinal applications.

Regarding antibacterial properties, parts of *M. balbisiana* show potential activity via inhibition of lots of bacterial strains. Parts of *M. balbisiana* are well known for their antibacterial properties. Tin et al. (2020) revealed that the inflorescence of *M. balbisiana* might be effective against bacterial strains of *Bacillus cereus, Vibrio parahaemolyticus, Listeria monocytogenes, Staphylococcus aureus, Yersinia enterocolytica*, and *Brohroctrix termosphacta*. The methanolic and ethanolic extracts showed more potent antibacterial activity than extracts in other solvents. The methanolic extract of *M. balbisiana* fruit showed strong antibacterial activity against *Shigella dysenteriae* with a zone of inhibition (ZOI) of 12–14 mm (Kusuma et al. 2017). Besides, the nanosynthesized leaf extract of *M. balbisiana* demonstrated potential bactericidal activity against *E. coli* and *S. aureus* with MICs of 1.0 and 2.0 nM, respectively (Maji et al. 2019). Bag et al. (2020) discovered that nanoparticles synthesized from *M. balbisiana* peel extract have the potential to inhibit bacteria. The 80% ethanolic extract from *M. paradisiaca* and *Musa acuminata* leaves inhibited the growth of *S. aureus* to resist Methicillin (MRSA) and susceptible *S. aureus* (MSSA). The results showed that the diameter of the inhibition zone of the ethanolic extracts of *M. paradisiaca* (2.86 g/mL) and *M. acuminata* (3.33 g/mL) had a similar antibacterial effect as the positive control of clindamycin—an antibiotic used to treat diseases related to MRSA. The extract from *M. sapientum* showed ineffectiveness on the tested bacteria (Sivasamugham et al. 2021). Thus, the extract of *Musa* sp. parts has antibacterial activity. However, it is necessary to compare the effectiveness of these parts.

Turning to the antifungal property, extract from pseudostem of *M. paradisiaca* AAB has the ability to inhibit 100% of the growth of *Aspergillus niger, Aspergillus oryzae*, and *Rhizopus stolonifer* strains at a concentration of 1.0 mg/mL. *M. paradisiaca* peel extract inhibits *A. niger* by 100%, *A. oryzae* by 76.67%, and *R. stolonifer* by 56.67% at the same concentrations (Okorondu et al. 2012). For *Candida albicans* strain, 50% ethanolic extract of *M. paradisiaca* peel gave an inhibition diameter of 12.5 mm, higher than the nystatin control (8.9 mm) (Loyaga‐Castillo et al. 2020). Besides, *M. paradisiaca* peel extract at 75% concentration decreased the amount of *C. albicans* as an acrylic denture cleaner (Fathiyyah et al. 2015). The methanolic extract of *M. acuminata* leaves showed positive effects towards *Staphylococcus epidermidis* and *Trichophyton mentagrophytes* (Harith et al. 2018). However, we have not found any studies evaluating and comparing the antifungal activity among *M. balbisiana* parts.

A number of researchers worldwide have investigated the antimicrobial and antifungal properties of plants. Also, several publications have reported about the antibacterial and antifungal activities of the individual parts of *Musa* species. However, there is a lack of studies investigating the comparison of antibacterial and antifungal abilities among *M. balbisiana* parts. Thus, this study aimed to evaluate and compare simultaneously: (i) the antibacterial activity as the minimum bactericidal concentration (MBC) and the minimal inhibitory concentration (MIC) assays; (ii) the antifungal properties of *M. balbisiana* parts. The findings from this study can partly elucidate the effectiveness of parts of *M. balbisiana* grown in Vietnam, and it offers essential information for further studies of applied parts of this plant in commercial antibacterial and antifungal products.

## 2 Materials and Methods

### 2.1 Materials

*M. balbisiana* collected from An Hoa ward, Tam Nong district, Dong Thap province, Vietnam (10°40′17″ N, 105°33′36″ E). Fresh corm, peel, fruit, inflorescence, and seed parts were cleaned, sliced, and dried at 60°C until under 10% moisture before grinding into powder (< 80 mesh size) and kept in zipper bags and stored at 4°C for all experiments (Hoang Thi Ngoc et al. 2024).

*Chemicals*: All chemicals and reagents used in the study at the analytical level originated from Merck and Sigma Aldrich.

*Bacteria strains*: *Staphylococcus aureus* ATCC 25923, *Bacillus subtilis* ATCC 6633, *Escherichia coli* ATCC 25922, *Salmonella enterica* ATCC 13076.

*Molds*: *Aspergillus niger* and *Aspergillus flavus*.

### 2.2 Methods

#### 2.2.1 Sample Preparation

The preparation for samples of *M. balbisiana* parts was conducted according to our previous study (Hoang Thi Ngoc et al. 2024). The samples were diluted with dimethyl sulfoxide (DMSO) to achieve different concentrations for the experiments.

#### 2.2.2 Antibacterial Activity

The antibacterial activity was assessed using the agar well diffusion method of minimum inhibitory concentration (MIC) and minimum bactericidal concentration (MBC) assays.

##### 2.2.2.1 Bacterial Strains Preparation

Four bacteria strains, including *S. aureus*, *B. subtilis*, *E. coli*, and *S. enterica*, were used. These bacteria strains were routinely maintained on Brain Heart Infusion (BHI) broth (Becton, Dickinson and Company, Sparks, MD) containing 25% glycerol (v/v) at −7°C and, when necessary, subcultured on a Tryptone Soya Broth (TSB) medium (Himedia, India). The cultures were incubated at 37°C for 1 day under ambient conditions. Then, these strains were inoculated into Mueller Hinton Agar (MHA, Himedia, India) for 24 h at 37°C to determine the number of colonies in the TSB enrichment medium. Next, bacterial suspensions containing 1 × 10^6^ CFU/mL were prepared by dilution.

##### 2.2.2.2 MBC Assay

Antibacterial activity in vitro was performed by determining the zone of inhibition according to the method described by Trieu Ly et al. (2021) with slight modifications. Overnight bacterial cultures were adjusted to a density of 1 × 10^6^–1 × 10^8^ CFU/mL, spread evenly on agar plates using sterile sticks. The samples of the corm, pseudostem, inflorescence, fruit, peel, and seed of *M. balbisiana* were impregnated on 6 mm‐diameter Whatman paper. The inhibition zone was measured on day 2 (measuring in mm). The inhibition zone (D: mm) was calculated using the formula:

(1) $$D=\left(D_{\text{sample}}–D_{\text{agar well}}\right)–\left(D_{\text{negative control}}–D_{\text{agar well}}\right)$$

Ampicillin and 10% DMSO were used as positive and negative controls, respectively.

##### 2.2.2.3 MIC Assay

The samples of the corm, pseudostem, inflorescence, fruit, peel, and seed of *M. balbisiana* were serially diluted in DMSO with the different ratios 1/2, 1/4, 1/8, and 1/16 (w/v) to gain various concentrations (6.25–800 μg/mL). The experiments were conducted in 96‐well plates. Each well was loaded with 50 μL of bacterial suspension (10^6^ CFU/mL) and 50 μL of samples at different dilutions. The plates were incubated at 37°C for 24 h. Following incubation, 30 μL of 0.01% resazurin solution (Acros Organics, Belgium) was added to each well and incubated again at 37°C for 2 h. The color change in each well was then observed. The minimum inhibitory concentration (MIC) was determined as the lowest sample concentration that maintained a blue color (indicating no bacterial growth) or the first dilution at which the color changed from blue to slightly purple (indicating notable growth inhibition) (Ngan et al. 2012). Control wells contained only the bacterial culture and medium, while amoxicillin was used as the positive control. All experiments were conducted in triplicate.

#### 2.2.3 Antifungal Activity

Mold preparation: molds ( *A. niger* and *A. flavus*) were inoculated into Petri dishes (90 mm, Germany) containing Potato Dextrose Agar (PDA) medium (Himedia, India) and incubated at 37°C for 5–7 days. Then, spores were collected and diluted in 0.9% saline solution (Mekophar, Vietnam) to obtain a concentration of 10^6^ spores/mL.

Antifungal activity was evaluated against the growth of *A. niger* and *A. flavus* according to the description of Tian et al. (2012) with minor modifications. Six different parts of *M. balbisiana* were dissolved in 10% dimethyl sulfoxide (DMSO) to obtain concentrations of 100, 200, 400, 800, 1600 μg/mL. 100 μL of the sample was spread onto a Petri dish containing a PDA medium. Then, 10 μL of mold spores was added to the center of each dish, and the plates were incubated at 28°C ± 2°C for 5 days. All tests were conducted in triplicate. Antifungal effectiveness was determined by measuring the diameter of fungal colonies, and the mold inhibition rate was calculated using the following formula:

(2) $$I\;\left(\%\right)=\left[\left(d_{c}-d_{t}\right)/d_{c}\right]\times 100$$

where, *d*_c_ (mm) is the average colony diameter of the control and *d*_t_ (mm) is the average colony diameter of the treated samples.

### 2.3 Data Analysis

All experiments were performed in triplicate, and the results are reported as mean ± standard deviation (SD). Statistical analysis was carried out using Minitab software, and differences between treatments were assessed using one‐way analysis of variance (ANOVA) at a significance level of *p* < 0.05.

## 3 Results and Discussion

### 3.1 Antibacterial Activity

#### 3.1.1 Antibacterial Activity via MBC Assay

Antibacterial activity of investigated samples via the MBC method was tested on four strains of pathogenic bacteria, including two gram‐negative strains (*Salmonella enterica, Escherichia coli*) and two gram‐positive strains ( *Staphylococcus aureus* , *Bacillus subtilis* ). Secondary metabolites' ability to inhibit these bacteria can be attributed to the differences in the cell wall structure of the four bacteria strains. Gram‐negative bacteria have a lipopolysaccharide layer with thin peptidoglycan, whereas gram‐positive bacteria have a thick peptidoglycan layer as part of their cell wall structure (Panagan and Syarif 2009).

**FIGURE 1:**
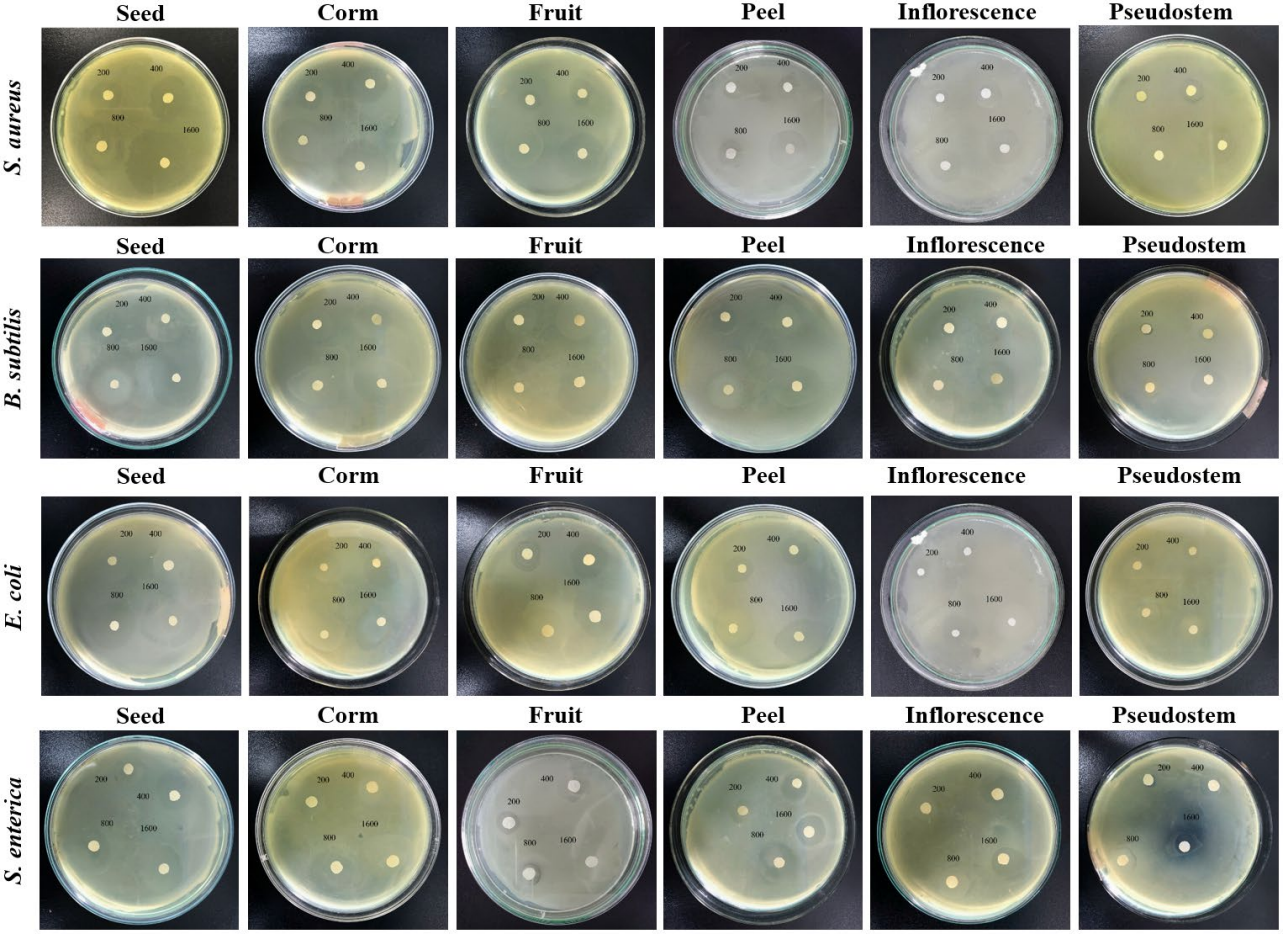
Antibacterial properties of samples from *Musa. balbisiana* parts on four selected bacterial strains.

**FIGURE 2:**
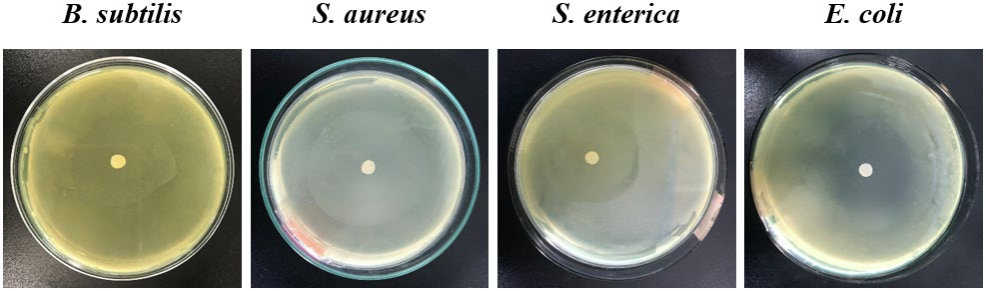
Antibacterial properties on four bacterial strains of the control (Ampicillin).

As shown in Table 1, Figures 1 and 2, the different parts of *M. balbisiana* demonstrated the ability to inhibit the growth of both gram‐positive and gram‐negative bacteria, with effectiveness rising in a concentration‐dependent manner. The diameter of the inhibition zones increased with an increase in sample concentration.

**TABLE 1:** Antibacterial properties of samples from *Musa balbisiana* parts.

Samples	Concentrations (μg/mL)	Antibacterial diameter (mm)			
		*Salmonella enterica*	*Escherichia coli*	*Staphylococcus aureus*	*Bacillus subtilis*
Seed	200	13.90 ± 0.45^d^	14.96 ± 0.30^D^	18.13 ± 0.35^dd^	15.16 ± 0.25^DD^
	400	16.46 ± 0.37^c^	17.30 ± 0.26^C^	24.33 ± 0.15^cc^	18.50 ± 0.36^CC^
	800	19.43 ± 0.30^b^	23.16 ± 0.35^B^	27.93 ± 0.41^bb^	25.03 ± 0.56^BB^
	1600	24.40 ± 0.20^a^	30.80 ± 0.40^A^	35.36 ± 0.30^aa^	33.56 ± 0.40^AA^
Corm	200	13.13 ± 0.65^d^	13.73 ± 0.15^D^	15.60 ± 0.36^dd^	14.00 ± 0.55^DD^
	400	15.10 ± 0.20^c^	16.50 ± 0.30^C^	21.86 ± 0.81^cc^	17.26 ± 0.15^CC^
	800	17.56 ± 0.20^b^	20.80 ± 0.96^B^	25.83 ± 0.70^bb^	21.23 ± 0.64^BB^
	1600	23.80 ± 0.26^a^	27.87 ± 0.81^A^	32.03 ± 0.76^aa^	28.10 ± 0.81^AA^
Fruit	200	11.43 ± 0.15^d^	12.23 ± 0.83^D^	15.73 ± 0.40^dd^	12.60 ± 0.30^DD^
	400	12.70 ± 0.20^c^	18.77 ± 0.38^C^	19.70 ± 0.66^cc^	14.36 ± 0.15^CC^
	800	15.66 ± 0.55^b^	22.27 ± 0.47^B^	24.27 ± 0.47^bb^	19.80 ± 0.55^BB^
	1600	21.50 ± 0.26^a^	26.80 ± 0.62^A^	30.33 ± 0.96^aa^	24.06 ± 0.37^AA^
Peel	200	10.76 ± 0.51^d^	11.83 ± 0.45^D^	11.93 ± 0.75^dd^	12.00 ± 0.70^DD^
	400	12.33 ± 0.25^c^	13.20 ± 0.81^C^	14.30 ± 0.20^cc^	13.7 ± 0.20^CC^
	800	14.16 ± 0.49^b^	15.56 ± 0.35^B^	17.60 ± 0.36^bb^	15.36 ± 0.96^BB^
	1600	19.63 ± 0.37^a^	20.43 ± 0.30^A^	22.46 ± 0.15^aa^	20.13 ± 0.45^AA^
Inflorescence	200	10.50 ± 0.19^d^	9.066 ± 0.58^D^	10.46 ± 0.25^dd^	9.80 ± 0.45^DD^
	400	11.93 ± 0.75^c^	11.53 ± 0.20^C^	14.30 ± 0.65^cc^	12.16 ± 0.66^CC^
	800	13.40 ± 0.26^b^	15.76 ± 0.55^B^	16.43 ± 0.41^bb^	16.23 ± 0.35^BB^
	1600	17.86 ± 0.35^a^	17.80 ± 0.17^A^	19.53 ± 0.37^aa^	18.30 ± 0.19^AA^
Pseudostem	200	7.733 ± 0.40^d^	8.30 ± 0.20^D^	8.23 ± 0.30^dd^	7.67 ± 0.55^DD^
	400	10.60 ± 0.26^c^	10.53 ± 0.15^C^	12.43 ± 0.25^cc^	10.40 ± 0.55^CC^
	800	13.06 ± 0.25^b^	11.46 ± 0.30^B^	15.90 ± 0.36^bb^	12.33 ± 1.00^BB^
	1600	15.70 ± 0.10^a^	13.40 ± 0.26^A^	18.13 ± 0.35^aa^	17.60 ± 0.29^AA^
Ampicilin		33.50 ± 0.36	37.70 ± 0.87	45.76 ± 0.41	41.36 ± 0.15

*Note:* For each sample, different letters indicate statistically significant differences (*p* < 0.05).

For the *B. subtilis* strain, at a concentration of 200 μg/mL, inflorescence and pseudostem samples showed minimal antibacterial activity at 200 μg/mL, with inhibition zone diameters of just 9.80 and 7.67 mm, respectively. The remaining samples showed weak antibacterial ability with diameters of 15.17 mm (seed), 14.00 mm (corm), 12.60 mm (fruit) and 12 mm (peel). At the highest concentration tested (1600 μg/mL), all samples showed markedly enhanced antibacterial effects. The inhibition zones increased to 33.57 mm (seed), 28.10 mm (corm), 24.06 mm (fruit), 20.13 mm (peel), 18.30 mm (inflorescence), and 17.60 mm (pseudostem).

Turning to the *S. aureus* , at a concentration of 200 μg/mL, the pseudostem sample showed negligible antibacterial activity at 200 μg/mL, with an inhibition zone of just 8.23 mm. The inflorescence, peel, and fruit samples indicated weak inhibition with diameters of 10.46, 11.93, and 15.73 mm, respectively. Antibacterial activity improved significantly at higher concentrations. At 800 μg/mL, the seed, corm, and fruit samples showed strong inhibition, with diameters of 27.93, 25.83, and 24.27 mm, respectively. In addition, this influence was further increased at 1600 μg/mL, reaching 35.37 (seed), 32.03 (corm), and 30.33 mm (fruit). The pseudostem, inflorescence, and peel samples demonstrated moderate antibacterial ability at these higher concentrations.

For *E. coli* , the investigated samples exhibited lower antibacterial activity compared to *S. aureus* and *B. subtilis* . At a concentration of 1600 μg/mL, the inhibition zone diameters were 30.80 (seed), 27.87 (corm), 26.80 (fruit), 20.43 (peel), 17.80 (inflorescence), and 13.40 mm (pseudostem).

Finally, the samples showed the lowest antibacterial ability on the *S. enterica* strain compared to the remaining bacteria strains. At a concentration of 1600 μg/mL, the inhibition zone diameters were observed in the following order: seed (24.40 mm), corm (23.80 mm), fruit (21.50 mm), peel (19.63 mm), inflorescence (17.87 mm), and pseudostem (15.70 mm).

The inconsistency in inhibition zone sizes may be attributed to the uneven distribution of bacteria on the Petri dish. Hence, the bacterial population does not grow densely in one place but grows densely in another place. Moreover, discs containing antibacterial agents are likely to form larger inhibition zones in areas with lower bacterial density than in areas where the bacteria are more densely concentrated (Panagan and Syarif 2009). Gram‐negative bacteria with differences in cell wall structures are generally more resistant to antibacterial agents than gram‐positive bacteria (Zuhud et al. 2001). However, a higher concentration of antibacterial compounds can enhance their capability to penetrate bacterial cells, disrupt metabolic functions, and induce cell lysis (Lingga et al. 2015).

The microbial cell membrane systems can be disrupted by saponins. According to Dong et al. (2020) saponins extracted from *Chenopodium quinoa* bark can cause severe bacterial damage by causing a degradation of the cell wall. Subsequently, the cytoplasmic membrane and membrane proteins are disrupted. The leakage of intracellular contents such as nucleic acids and proteins, which are key components that typically remain confined within healthy cells, is a result of membrane dysfunction caused by this damage. When these molecules appear outside the cell, it suggests that membrane breakdown has taken place and eventually causes bacterial cell death. Manso et al. (2021) reported that a variety of polyphenols, specifically flavonoids, display substantial antibacterial effects against clinical bacterial isolates. These include flavonols such as morin, quercetin, and kaempferol, as well as flavonols and their derivatives, such as (−)‐epigallocatechin gallate, (+)‐acyl catechin derivatives, epicatechin gallates, 3‐O‐decyl‐(+)‐catechin, and (+)‐catechin. Also included are phenolic acids and their derivatives, which include protocatechuic acid, ethyl ester, and caffeic acid (Manso et al. 2021). Enormous structural polyphenolic compounds result in functional diversity. The active polyphenols, such as flavonoids or hydrolyzable tannins, could be effective against bacteria. Their antibacterial properties can be manifested in three ways: directly killing bacteria, synergistically activating antibiotics, and attenuating bacterial pathogenicity. Furthermore, flavonoids have demonstrated their capacity to inhibit the efflux pump and destabilize the cytoplasmic membrane. They have the ability to prevent antibiotic resistance in bacteria by inhibiting β‐lactamases and topoisomerase (Xie et al. 2017).

The study by Saro et al. (2022) found that the inhibition zones for *S. aureus* were similar for both Lacatan ( *M. acuminata* C.) and Cardaba (*M. acuminata balbisiana*), with measurements of 15.35 ± 2.13 mm and 15.30 ± 3.72 mm, respectively, suggesting little difference in antibacterial activity (Saro et al. 2022). Parvez et al. (2020) reported that the inedible part of unripe *Musa sapientum* fruit showed stronger antibacterial activity on *Salmonella* sp. (16.2 mm) than the edible part (14.2 mm) at a concentration of 800 mg/mL.

Likittrakulwong et al. (2023) studied the antibacterial activities of peels from *M. acuminata* , *M. sapientum* , and *M. balbisiana* . Among them, *M. sapientum* indicated the strongest antibacterial ability with the inhibition diameters of *B. subtilis* (13 mm), *S. aureus* (15 mm), and *E. coli* (13 mm). In contrast, *M. acuminata* showed the lowest antibacterial property against all investigated microbial strains. Onyema et al. (2016) reported that the highest diameter of the antibacterial zone of water extract from *M. acuminata* pseudostem was 21 mm for *Streptococcus* sp. compared to the antibacterial zone (14 mm) of the methanolic extract. However, the water extract did not inhibit *S. aureus* and *E. coli* so no inhibition zones were seen.

In summary, all parts of *M. balbisiana* showed potential inhibition of gram‐positive bacteria better than gram‐negative bacteria in the MBC assay. Besides, the seed sample indicated the highest antibacterial activities among the six selected parts.

#### 3.1.2 Antibacterial Activity via MIC Method

**FIGURE 3:**
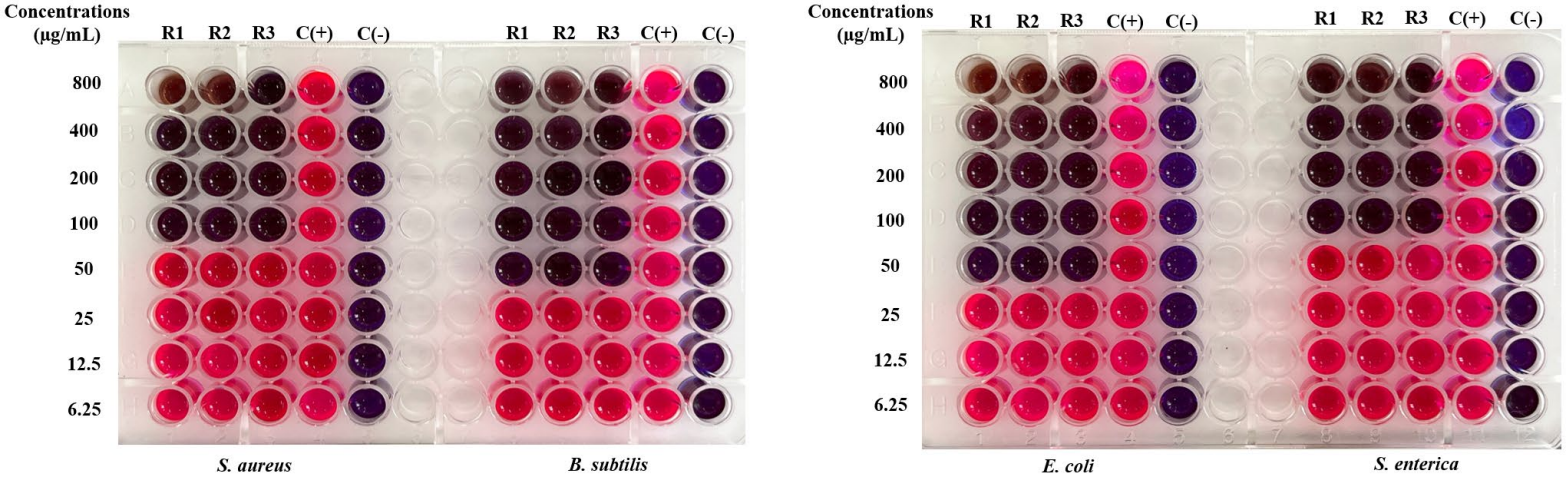
The antibacterial activity of seed sample via MIC assay.

**FIGURE 4:**
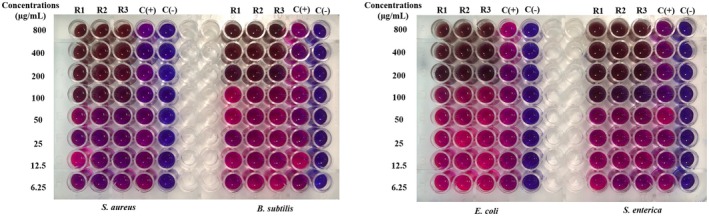
The antibacterial activity of corm sample via MIC assay.

**FIGURE 5:**
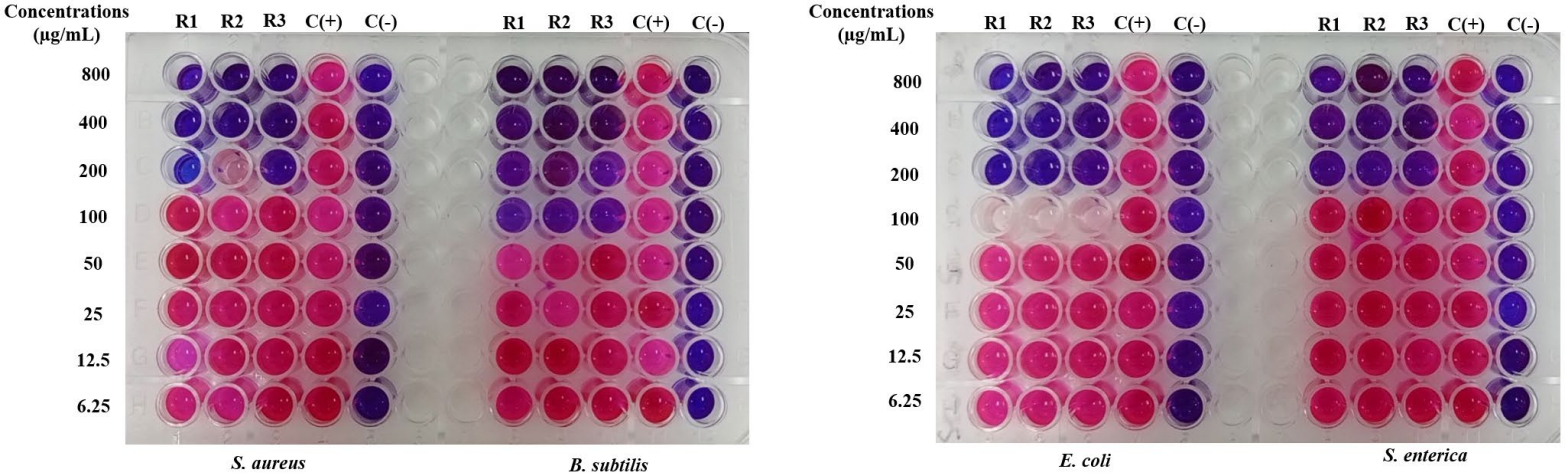
The antibacterial activity of fruit sample via MIC assay.

**FIGURE 6:**
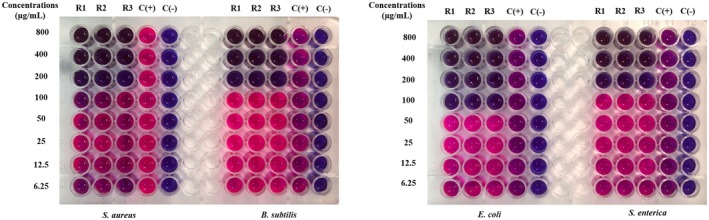
The antibacterial activity of peel sample via MIC assay.

**FIGURE 7:**
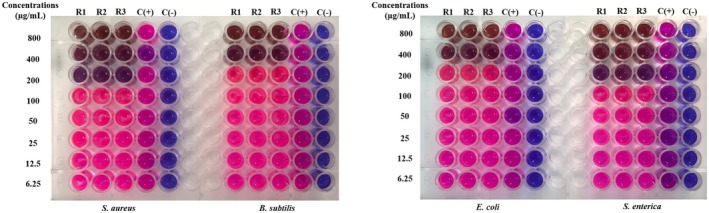
The antibacterial activity of inflorescence sample via MIC assay.

**FIGURE 8:**
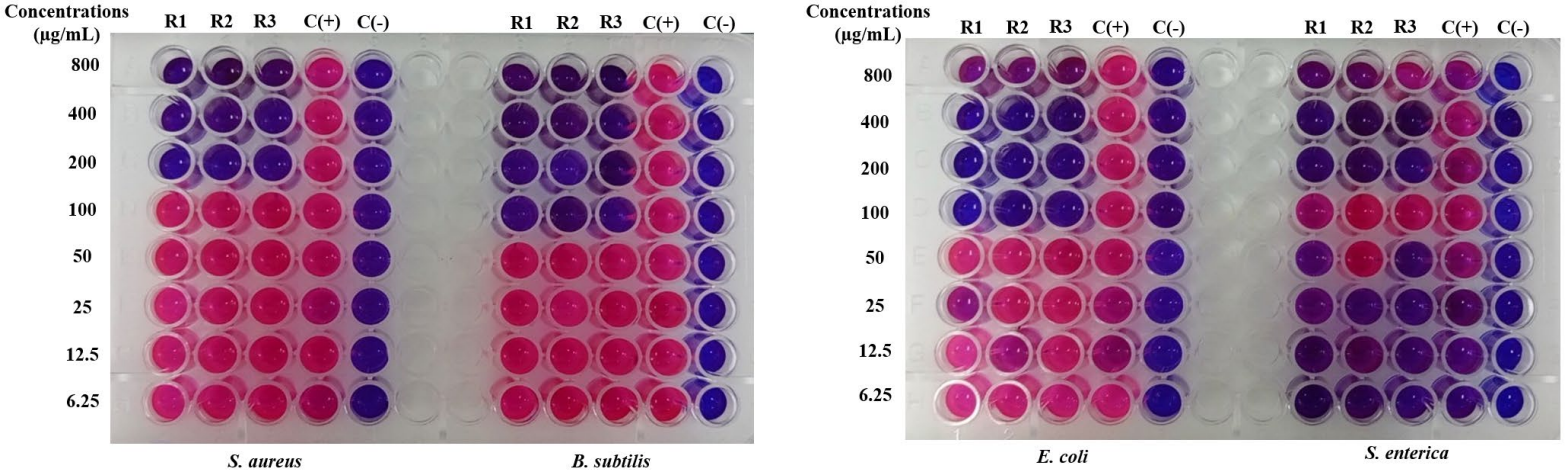
The antibacterial activity of pseudostem sample via MIC assay. Noticed: C(+): positive control; C(−): negative control; R1, R2, and R3: the experiments were repeated the first, second, and third times.

The MIC value is the lowest concentration of the sample, which means that the bacteria are completely inhibited from growth. It is determined as the lowest concentration where bacterial growth is reduced by more than 80% compared to the positive control. To facilitate result interpretation, resazurin, a blue dye with weak fluorescence, is used. Active bacteria convert resazurin into the fluorescent pink compound, resorufin. The MIC results for *M. balbisiana* samples are presented in Figures 3, 4, 5, 6, 7, 8.

Wells that showed no color change in the resazurin reagent (remaining blue) indicated the concentration at which bacterial growth was fully inhibited. In contrast, wells that turned pink signified bacterial growth. Figure 3 reveals that the seed sample showed the MIC value at 100 μg/mL for *S. aureus* and *S. enterica* , and at 50 μg/mL for two strains, *B. subtilis* and *E. coli* . Besides, the corm sample had a MIC value of 100 μg/mL for *S. aureus* and *S. enterica* strains, while *B. subtilis* and *E. coli* strains had a MIC value of 200 μg/mL (Figure 4). The fruit sample showed a MIC value of 100 μg/mL for both *S. aureus* and *S. enterica* , while the other two strains showed weaker inhibition at 200 μg/mL (Figure 5). The MIC value of the peel sample to two bacterial strains, B. subtilis and S. enterica, was 200 μg/mL, while stronger inhibition was observed for *S. aureus* and *E. coli* at MIC 100 μg/mL (Figure 6). *M. balbisiana* inflorescence sample showed weaker antibacterial ability on all four chosen bacterial strains overall. The results from Figure 7 show that the *M. balbisiana* inflorescence sample had high inhibitory ability against *S. aureus* and *S. enterica* strains (MIC 200 μg/mL) and also against two strains, *E. coli* and *B. subtilis* , which showed lower inhibition with a MIC value of 400 μg/mL. Next, the pseudostem sample showed no color change in wells at concentrations ranging from 200 to 800 μg/mL, indicating a MIC of 200 μg/mL for *S. aureus* and *S. enterica* , and 100 μg/mL for *B. subtilis* and *E. coli* (Figure 8).

The antibacterial effects of *M. balbisiana* parts in this study were notably stronger than those reported in several earlier studies. Compared with the findings of Venkatesh et al. (2013) MIC of ethanoic extract from the corm of *M. paradisiaca* and *Musa acuminate* was 1.5 mg/mL and 2 mg/mL for both strains of *S. aureus* and *B. subtilis* , respectively. As for *the E. coli* strain, the MIC values were even higher at 4 mg/mL and 3 mg/mL. In another study, Ismail et al. (2018) reported that acetone extract from the pseudostem of *M. acuminata* inhibited *P. aeruginosa* and *E. coli* at MICs of 125 mg/mL and 250 mg/mL, respectively. The lipopolysaccharide (LPS) layer of gram‐negative bacteria is used to protect them, preventing the inner membrane from being exposed to its natural antibacterial activities. Compared to gram‐positive bacteria, gram‐negative bacteria are often more resistant to plant‐based antibiotics and are not affected. Muhammad Mirza et al. (2021) assessed the antibacterial activity of extracts from *M. paradisiaca* inflorescence using both MIC and MBC methods. All four extracts, hexane, chloroform, ethyl acetate, and methanol, demonstrated weak antibacterial activity against *S. aureus* and *S. pyogenes* with a MIC value of 450 μg/mL. The results in the MBC method also were similar, indicating comparable inhibitory effects. Additionally, Bui T.N. et al. tested the antibacterial activity of *M. balbisiana* seed extracts on *Enterococcus faecalis* and *S. aureus* via the MIC method. The ethyl acetate (EtOAc) extract exhibited the most potent activity, with MIC values of 7.5 mg/mL for *E. faecalis* and 3.75 mg/mL for *S. aureus* . However, petroleum extracts showed no activity in the concentration range of 0.02–10 mg/mL, suggesting an MIC higher than 10 mg/mL. The butanol extract exhibited an MIC of 22.5 mg/mL against both bacterial strains. Similarly, *M. sapientum* seed extract in EtOAc also had the best antibacterial activity against *S. aureus* compared to hexane and ethanol extracts (Bui et al. 2016). In another study, the ethanolic extract of *M. sapientum* peel also exhibited MIC ranging from 16 mg/mL to 512.5 mg/mL. The lowest MIC was 16 mg/mL for *S. typhi*, while *B. subtilis* and *S. aureus* had the highest MIC of 512.5 mg/mL. The MIC and MBC values of the aqueous extract ranged from 0 to 1025 mg/mL and 0 to over 1025 mg/mL, respectively. The water extract showed MIC of 64 mg/mL ( *E. coli* ) and 1025 mg/mL ( *B. cereus* ) (Ehiowemwenguan et al. 2014). Moreover, the findings in the report of Asoso et al. (2016) confirmed that the MIC value was 200–300 mg/mL for *M. paradisiaca* fruit extracts and MIC was 100–300 mg/mL (peel extract) on strains of *E. coli* (clinical and typed isolates) and *Shigella dysentrariae* (ATCC 24162) *Bacillus subtilis* (ATCC 21332).

In short, the *M. balbisiana* seed sample showed the best antibacterial activity on four investigated bacteria strains, which was indicated via MIC values.

### 3.2 Antifungal Activity

**FIGURE 9:**
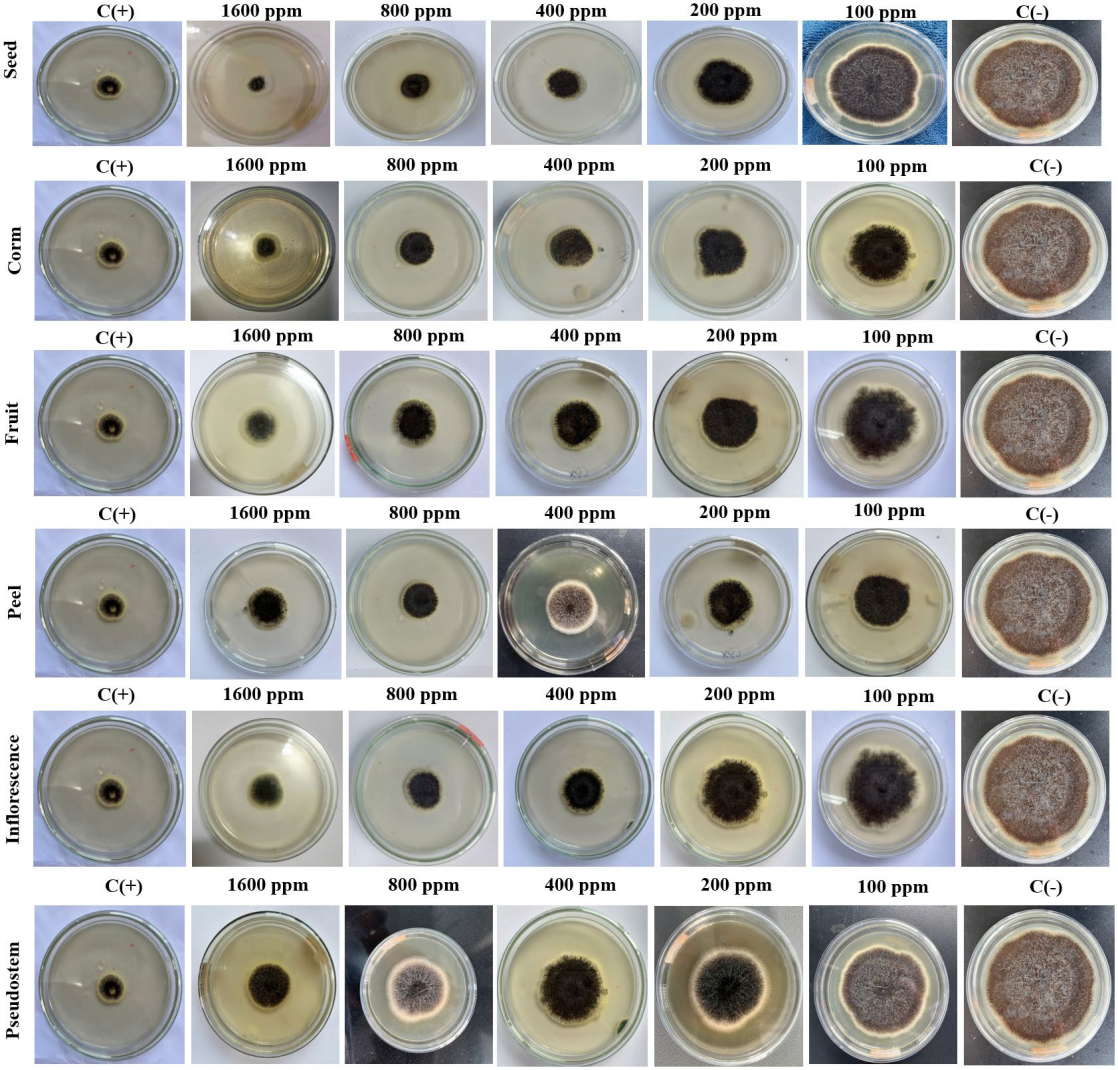
The effects of samples from *Musa. balbisiana* parts on the growth diameter of *Aspergillus niger*.

**FIGURE 10:**
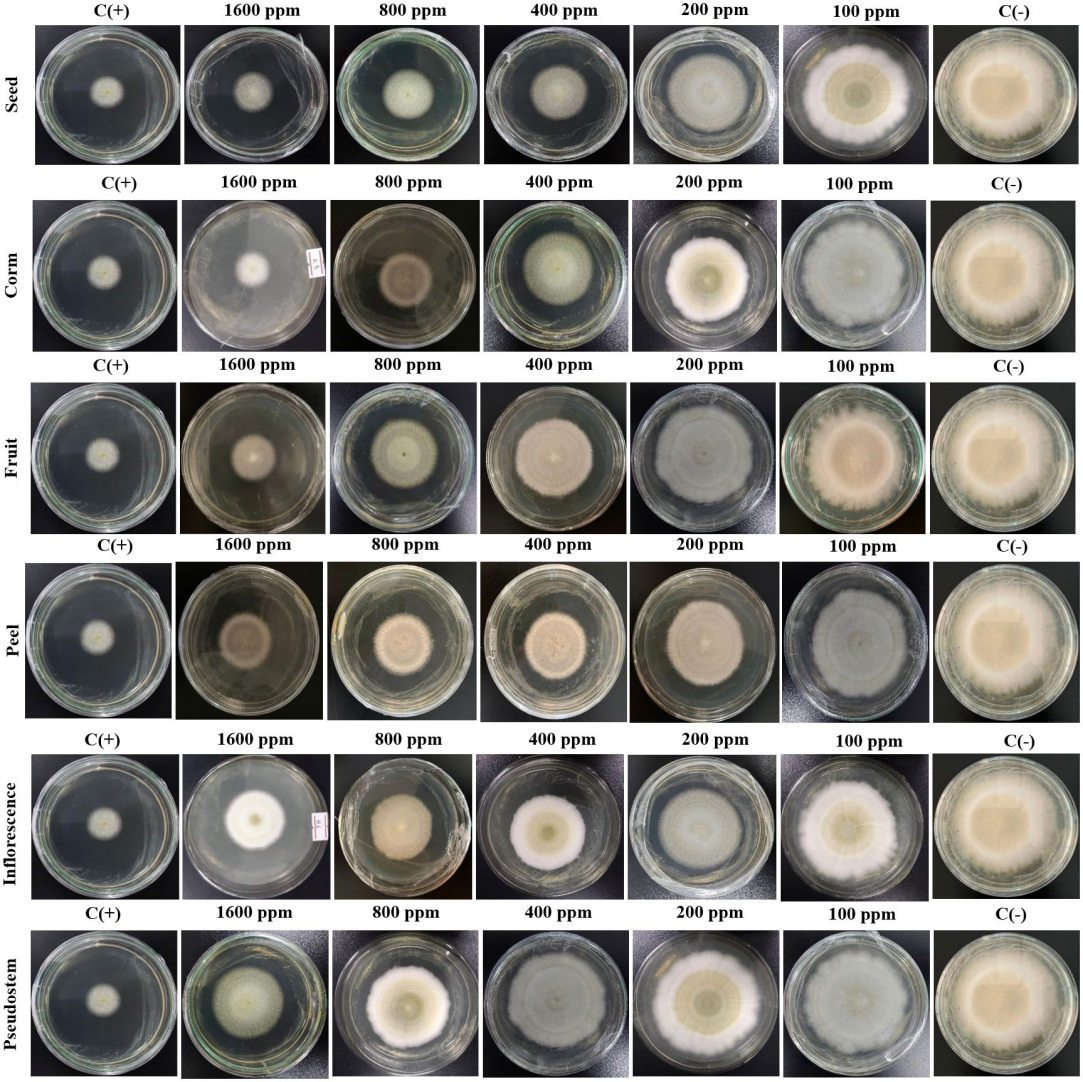
The effects of samples from *Musa balbisiana* parts on the growth diameter of *Aspergillus flavus*. Noticed: C(+): positive control; C(−): negative control.

In recent years, there has been a growing interest in research on natural products and plant‐derived compounds that possess strong antifungal qualities, mainly because of their significance in the development of functional foods and pharmaceuticals. Secondary compounds such as saponins, polyphenols, and flavonoids tend to be stored in plant cells as precursors that are not yet active. When pathogens attack, enzymes can quickly convert these compounds into biologically active forms that act as natural antibiotics. Their antifungal properties are commonly linked to the destruction of fungal cell membrane structure (Arif et al. 2009). The experimental results of the antifungal activity of *M. balbisiana* parts on *A. niger* and *A. flavus* are shown in Table 2, Figures 9 and 10.

**TABLE 2:** The inhibition ability of *Aspergillus niger* and *Aspergillus flavus* of samples from *Musa balbisiana* parts.

Samples	Concentrations (μg/mL)	Diameter inhibition (mm)		Percent inhibition (I%)	
		*Aspergillus flavus*	*Aspergillus niger*	*Aspergillus flavus*	*Aspergillus niger*
Seed	100	54.40 ± 0.69^a^	68.76 ± 0.56^A^	15.09 ± 1.11^aa^	17.80 ± 1.12^AA^
	200	47.13 ± 0.72^b^	55.76 ± 1.30^B^	26.43 ± 0.76^bb^	33.34 ± 1.89^BB^
	400	38.63 ± 1.11^c^	49.03 ± 0.41^C^	39.70 ± 1.61^cc^	41.39 ± 0.97^CC^
	800	23.40 ± 0.29^d^	23.16 ± 0.55^D^	63.47 ± 0.62^dd^	72.31 ± 0.43^DD^
	1600	15.56 ± 0.70^e^	17.40 ± 0.45^E^	75.70 ± 1.01^ee^	79.20 ± 0.53^EC^
Corm	100	55.40 ± 1.60^a^	72.56 ± 0.96^A^	13.53 ± 2.09^aa^	13.26 ± 1.17^AA^
	200	42.83 ± 0.35^b^	65.80 ± 2.09^B^	33.14 ± 0.38^bb^	21.35 ± 2.82^BB^
	400	37.60 ± 0.79^c^	50.83 ± 0.35^C^	41.31 ± 0.95^cc^	39.24 ± 0.53^CC^
	800	29.93 ± 1.19^d^	38.26 ± 1.83^D^	53.27 ± 2.10^dd^	54.26 ± 2.29^DD^
	1600	17.13 ± 0.35^e^	26.50 ± 1.21^E^	73.25 ± 0.66^ee^	68.33 ± 1.40^EE^
Fruit	100	53.93 ± 1.19^a^	67.43 ± 0.15^A^	15.80 ± 1.59^aa^	20.80 ± 1.74^AA^
	200	39.50 ± 0.20^b^	59.70 ± 0.19^B^	38.34 ± 0.47^bb^	36.21 ± 0.89^BB^
	400	27.63 ± 0.75^c^	45.36 ± 0.61^C^	56.86 ± 1.35^cc^	47.60 ± 2.23^CC^
	800	21.16 ± 0.35^d^	32.50 ± 0.81^D^	66.96 ± 0.39^dd^	55.21 ± 1.23^DD^
	1600	17.80 ± 0.36^e^	27.20 ± 1.21^E^	72.21 ± 0.56^ee^	67.72 ± 1.17^EE^
Peel	100	53.83 ± 0.89^a^	66.26 ± 1.93^A^	15.97 ± 0.99^aa^	19.40 ± 0.85^AA^
	200	45.90 ± 0.69^b^	53.36 ± 0.66^B^	38.34 ± 0.47^bb^	28.64 ± 0.73^BB^
	400	41.63 ± 1.06c	43.83 ± 1.51^C^	56.86 ± 1.35^cc^	45.77 ± 1.10^CC^
	800	35.40 ± 0.43^d^	37.46 ± 0.86^D^	59.67 ± 1.52^dd^	61.16 ± 0.82^DD^
	1600	21.10 ± 0.43^e^	27.00 ± 0.75^E^	65.40 ± 1.43^ee^	67.49 ± 1.35^EE^
Pseudostem	100	56.60 ± 1.13^a^	74.63 ± 0.55^A^	11.65 ± 1.33^aa^	15.90 ± 1.26^AA^
	200	54.16 ± 0.81^b^	64.50 ± 1.90^B^	15.45 ± 1.13^bb^	22.46 ± 1.07^BB^
	400	45.56 ± 0.35^c^	58.30 ± 1.30^C^	28.87 ± 0.88^cc^	38.37 ± 1.17^CC^
	800	41.33 ± 0.47^d^	43.96 ± 1.09^D^	35.48 ± 0.96^dd^	43.11 ± 1.43^DD^
	1600	30.53 ± 0.60^e^	37.60 ± 1.27^E^	52.34 ± 1.13^ee^	61.72 ± 0.71^EE^
Inflorescence	100	57.56 ± 0.75^a^	70.36 ± 1.30^A^	10.14 ± 1.59^aa^	10.79 ± 0.63^AA^
	200	46.86 ± 0.65^b^	64.86 ± 0.35^D^	26.84 ± 1.31^bb^	22.92 ± 1.65^BB^
	400	39.43 ± 1.10^c^	51.56 ± 1.15^C^	38.44 ± 1.76^cc^	30.32 ± 1.11^CC^
	800	32.10 ± 1.08^d^	47.60 ± 1.22^D^	49.89 ± 1.54^dd^	47.44 ± 1.73^DD^
	1600	30.66 ± 1.62^e^	32.03 ± 0.77^E^	52.13 ± 2.41^ee^	55.05 ± 1.81^EE^

*Note:* For each sample, different letters indicate statistically significant differences (*p* < 0.05).

According to Table 2, Figure 9 and Figure 10, different parts of *M. balbisiana* demonstrated inhibitory effects on the growth of *A. niger* and *A. flavus* . At the lowest tested concentration (100 μg/mL), the antifungal activity was minimal for all samples. Specifically, the inhibition percentages were as follows: seeds ( *A. niger* : 17.80%; *A. flavus* : 15.09%), corm ( *A. niger* : 13.26%; *A. flavus* : 13.53%), fruit ( *A. niger* : 20.80%; *A. flavus* : 17.84%), peel ( *A. niger* : 19.40%; *A. flavus* : 15.97%), pseudostem ( *A. niger* : 15.90%; *A. flavus* : 11.65%), inflorescence ( *A. niger* : 10.79%; *A. flavus* : 10.14%). The diameter of the mold colony was consistently reduced as the sample concentration increased from 200 μg/mL to 1600 μg/L, indicating significant improvement in antifungal efficacy. All *M. balbisiana* samples tested at 1600 μg/mL showed significant antifungal activity towards *A. niger* . The seed sample showed the most powerful influence, with a 79.20% reduction in fungal growth and a diameter of 17.40 mm. Similarly, the inhibitory ability and growth diameter were 68.33% and 26.50 mm (corm), 67.72% and 27.20 mm (fruit), 67.49% and 27.00 mm (peel), 61.72% and 37.60 mm (inflorescence) and 55.05% and 32.03 mm (pseudostem) (Figure 9). With *A. flavus* , the inhibitory ability and a growth diameter at 1600 μg/mL were 75.70% and 15.57 mm (seed), 73.25% and 17.13 mm (corm), 72.21% and 17.80 mm (fruit), 65.40% and 21.10 mm (peel), 52.34% and 30.53 mm (pseudostem), and 52.13% and 30.66 mm (inflorescence) (Figure 10). These results indicate that increasing sample concentrations enhance the antifungal activity of saponins and polyphenols. Their molecular size and functional groups likely influence the antifungal properties of polyphenols. These compounds have been proposed to support antifungal effects through multiple mechanisms. Fungal cell walls become deformed when glycan and chitin biosynthesis is inhibited, and when the cytoplasmic membrane is disrupted or its biosynthesis is disrupted, intracellular components leak out. This leads to the inhibition of fungal nucleic acid metabolism through the inhibition of mitochondrial processes and metabolic enzymes (Liu et al. 2021). The antifungal mechanism of phenolic compounds is largely attributed to their structural features, particularly the presence of hydroxyl (–OH) and carbonyl (C=O) groups. The fungal cell membrane is constituted of protein that phenolic compounds can damage due to their OH groups forming hydrogen bonds with N and H atoms in a protein of membrane cells. As a result, it damages the nutrient transport pathways, ultimately leading to toxic effects on fungi. However, *M. balbisiana* parts exhibit antifungal properties that are not solely attributed to their phenolic compounds. The fungus' growth is properly inhibited by multiple compounds present in the sample due to their synergistic effect (Mashuni et al. 2023).

In addition, saponin compounds exhibit antibacterial activity based on their ability to form complexes with sterols present in the membranes of microorganisms. This causes membrane damage and consequent cell collapse (Barile et al. 2007). Prakash et al. (2017) reported that both the powder and ash of *M. paradisiaca* peel were effective against *A. niger* with an inhibition zone of 26 mm. Similarly, Adamu et al. (2021) revealed that the antifungal activity of the flavonoid fraction of *Hyptis spicigera* leaf extractinhibited *A. flavus* with the growth inhibition zone, and no inhibition zone was found ( *A. niger* ). Rattan et al. (2017) demonstrated that isolated triterpenoid saponins had anti‐mold activity on *Lepidagathis cuspidata*. In detail, crude saponins and two isolated compounds showed antifungal potential in the inhibition range of 4–6 mm for *A. flavus* , *Penicillum nodatum*, and *Aspergillus fumigatus*.

In short, among all tested plant parts, the seed sample of *M. balbisiana* showed the most potent antifungal activity, resulting in the highest inhibition percentages against *A. niger* (79.20%) and *A. flavus* (75.70%) at the concentration of 1600 μg/mL.

## 4 Conclusion

This study is the first to simultaneously determine the antibacterial and antifungal activities of different *M. balbisiana* parts, including the corm, pseudostem, inflorescence, fruit, peel, and seed. The findings indicate that the selected parts possess significant antimicrobial properties, which are demonstrated via MIC and MBC assays against *S. aureus* , *S. enterica* , *B. subtilis* , and *E. coli* , as well as antifungal assays against *A. niger* and *A. flavus* . The antibacterial and antifungal activities were found to be concentration‐dependent, with the seed sample indicating the strongest effects for both antibacterial and antifungal tests. These findings are promising; it is important to note that these are in vitro experiments. Thus, further in vivo research is necessary to figure out the efficacy, safety, and optimal dosages of *M. balbisiana* parts for particular antibacterial or antifungal products to gain highly efficient treatment.

## Author Contributions

**Hoang Thi Ngoc Nhon:** conceptualization (lead), investigation (lead), methodology (lead), writing – review and editing (lead). **Dinh Khanh Dieu:** investigation (equal), methodology (equal). **Dong Thi Anh Dao:** conceptualization (equal), writing – review and editing (equal). **Le Thi Hong Anh:** conceptualization (equal), writing – review and editing (equal).

## Conflicts of Interest

The authors declare no conflicts of interest.

## Data Availability

The corresponding author is willing to share the data supporting the findings of this study upon reasonable request.
